# Joint Covariate Detection on Expression Profiles for Identifying MicroRNAs Related to Venous Metastasis in Hepatocellular Carcinoma

**DOI:** 10.1038/s41598-017-05776-1

**Published:** 2017-07-13

**Authors:** Xudong Zhao, Lei Wang, Guangsheng Chen

**Affiliations:** Northeast Forestry University, College of Information and Computer Engineering, Harbin, 150001 China

## Abstract

Expression profiles of cancer are generally composed of three dimensions including gene probes, patients (e.g., metastasis or non-metastasis) and tissues (i.e., cancer or normal cells of a patient). In order to combine these three dimensions, we proposed a joint covariate detection that not only considered projections on gene probes and tissues simultaneously, but also concentrated on distinguishing patients into different groups. Due to highly lethal malignancy of hepatocellular carcinoma, we chose data GSE6857 to testify the effectiveness of our method. A bootstrap and accumulation strategy was introduced in, which could select candidate microRNAs to distinguish metastasis from non-metastasis patient group. Two pairs of microRNAs were further selected. Each component of either significant microRNA pair was derived from different cliques. Targets were sought and pathway analysis were made, which might reveal the mechanism of venous metastasis in primary hepatocellular carcinoma.

## Introduction

Globally, hepatocellular carcinoma (HCC) is a common and highly lethal malignancy. It has been generally accepted that the invasive and metastatic potentials of HCC are mostly attributed to rapid recurrence and poor survival of HCC^1^. Therefore, identifying molecules that can suppress metastasis may provide novel targets for HCC therapies. MicroRNAs (miRNAs) are a class of highly conserved short RNAs that regulate diverse cellular processes by binding to the 3′untranslated region (3′-UTR) of target messenger RNAs (mRNAs)^2^. To date, several miRNAs have been characterized to have proangiogenic (miR-221^3^) or antiangiogenic (miR-122^4^, miR-29b^5^ and miR-214^6^) activities or to possess prometastatic (miR-151^7^, miR-30d^8^, miR-210^9^ and miR-135a^10^) or antimetastatic (miR-122^11^, miR-124^12^, miR-139^13^, miR-125b^14^, miR-29b^5^ and miR-7^15^) functions in HCC. Therefore, miRNAs could serve as therapeutic targets in HCC.

Different miRNAs associated with HCC were derived due to various statistical methods used for screening on genome-wide expression profiles. Note that expression profiles are composed of features or variables (e.g. miRNAs and mRNAs) in row, each of which is across different samples or patients. Commonly, a univariate paired t-test was performed to identify significant miRNAs for discrimination of two groups such as cancer and normal tissues^16^, virus and non-virus patients^17^, vascular invasion and primary HCC specimens^18^, and metastasis and non-metastasis samples^19, 20^. Besides, a multivariate t-test with permutations of group labels was provided for identification of miRNAs associated with HCC metastasis^21^. Methods mentioned above were also used to establish gene signatures for HCC metastasis^22^ or HCC recurrence^23, 24^.

In fact, these obtained statistical significances are faced with three major problems. First, prevailing studies mainly extracted individual features regardless of their coordination. It was reported that additions or subtractions of expression values from two individually selected miRNAs were provided for a better discriminative performance^20^. However, it has been indicated that two individual features, each of which is differentially expressed, may not correspond to the pair with a best discriminative performance^25^. Second, most of existing methods treated cancer and adjacent normal tissues separately. As far as HCC metastasis is concerned, statistical analysis was made either only on HCC tissues^19, 20, 22^ or on HCC and adjacent normal tissues respectively^21^. A certain combination of cancer and adjacent normal expressions is to be made so that a better discriminative performance of selected features between two groups can be justified. Third, most of feature selection methods were based on hypothesis testing, which aimed to evaluate whether two populations of samples were significantly different or not by a certain discriminative statistics. On the contrary, classification that aimed at classifying samples into the right population they belong to was only viewed as a posterior validation of features selected by anterior hypothesis testing.

On the basis of these insights, we proposed a joint covariate detection method that combined not only cancer and adjacent normal expression profiles but also hypothesis testing and classification methods. First, individual features and feature tuples on expression profiles were simultaneously taken into account. Considering a large amount enumeration of feature tuples, we only performed up to feature pairs for simplicity. Second, a linear projection on cancer and adjacent normal expressions of each feature was made. Commonly, expressions of each feature were regarded as a basic sampling unit in a vector form. That is, the expressions of each sample on this feature formed a two-dimension vector with its two components representing cancer and normal tissue respectively. Regardless of the correlation among features, Fisher’s linear discriminative analysis (LDA) was made on each feature with corresponding tumor and adjacent non-tumor expressions projected onto a most discriminative orientation for differentiating between different patients (e.g., metastasis and non-metastasis). As to each feature pair, expressions from cancer and adjacent normal tissues were viewed as a matrix form. That is, the expressions of each sample on each feature pair formed a second-order matrix, which is composed of two column vectors derived from the the cancer and normal tissue of each feature. Accordingly, a bilinear form and matrix-variate discriminative analysis^26^ were provided. In view of the limited sample size that led to a poor covariance matrix of each group, we presented an approximate implementation for simplicity. Third, the thought of integrative hypothesis testing (IHT)^27^ was introduced in. To be specific, we developed Fisher’s LDA-based classification together with Welch’s t-test to be an IHT for coordinative selection of individual or pairwise feature candidates. We implemented the joint covariate detection approach on miRNA expression profiles of primary HCCs publicly available at the gene expression ominbus (GEO) with its accession number GSE6857^21^, and ultimately extracted two miRNA pairs that might be associated with HCC venous metastasis. Potential target genes of these miRNAs were selected using TarBase^28^ and the corresponding KEGG pathway was selected using DAVID^29^, which testified the significance of the selected miRNAs.

## Results

**Figure 1:**
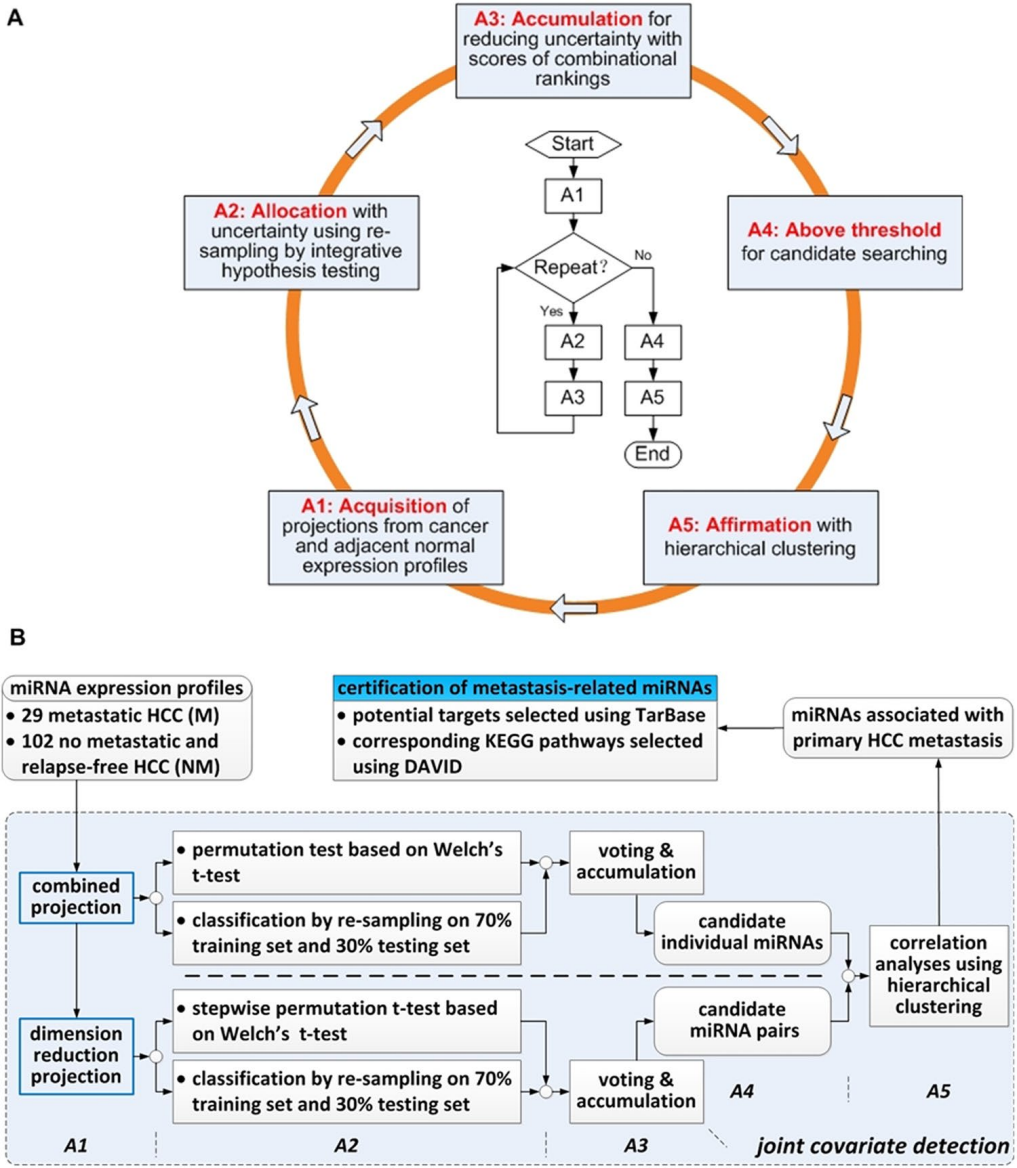
Selection schematic of metastasis-related miRNAs. Panel (A) represents a bilinear A5-based feature selection method. Panel (B) denotes the corresponding joint covariate detection for identifying metastasis-related miRNAs in HCC.

On assumption that expression profiles are composed of three dimensions including feature (e.g., miRNA or mRNA), sample (i.e., metastasis or non-metastasis) and tissue (i.e., cancer specimen or normal specimen), joint covariate detection embodies not only a bilinear projection on tissue and feature dimension, but also IHT on sample dimension. Besides, an A5 formulation^30^ was emulated in order to overcome the problem of small sample size compared with large feature numbers. As illustrated in Fig. 1A, A5 performed enough times of re-sampling, made a linear projection and a bilinear matrix-variate projection at step A1, unified combinational rankings from IHT at step A2, accumulated the scores of all the times at step A3 for candidate selection at step A4, and ultimately made an affirmation with hierarchical clustering at step A5. The corresponding joint covariate detection method on HCC was illustrated in Fig. 1B.

### Linear projection, bilinear projection and approximate implementation at A1 step

In order to discover metastasis-related miRNAs differentially expressed in HCC, 131 primary HCC patients with metastasis or non-metastasis cases were confirmed from a public dataset with its accession number GSE6857^21^. Among them, 29 samples were associated with metastasis cases. The other 102 samples corresponded to non-metastasis cases. Missing values of the downloaded normalized data were imputed using k nearest-neighboring algorithm in Euclidean distance. Probes associated with human miRNAs were extracted for further analysis. We utilized the A5-based feature selection method for searching individuals and pairs of probes expressed differently between metastatic and metastatic-free HCC group.

Above all, a combination needed to be made between HCC and adjacent normal expression profiles of each individual probe for a better discriminative performance. Therefore, Fisher’s LDA which kept the smallest variance within each phenotype group (i.e., metastatic or not) and provided a most discrimination between two groups was utilized. As to each probe pair, expressions of HCC and adjacent normal tissues formed a three-dimensional matrix, of which the dimensions were along the probe pair, two phenotype groups of patients (i.e., metastasis and metastasis-free HCCs) and HCC accompanied with its adjacent normal tissues. Xu proposed a bilinear form and matrix-variate discriminative analysis of microarrays^26^, which were displayed in detail from Equations (41) to (43) in that paper. Here, we presented an approximate implementation. This simplification converted the bilinear form to two separated learning steps on projection directions of not only the HCC accompanied with its adjacent normal tissues but also each pair of probes. In other words, the bilinear form corresponded to a two-step Fisher’s linear projection, i.e., combined projection and dimension reduction projection, as shown in Fig. 1B. Combined projection represented a linear projection of cancer and adjacent normal expressions on each individual feature. As to dimension reduction projection, it corresponded to a secondary linear projection between the combined projection results of each feature pair. We firstly made combined projection with the component of HCC to be positive in the orientation of projection using all 131 samples, as shown in A1 module of Fig. 1A.

### Cyclic A2 and A3 for obtaining accumulated scores of combinational rankings

**Figure 2:**
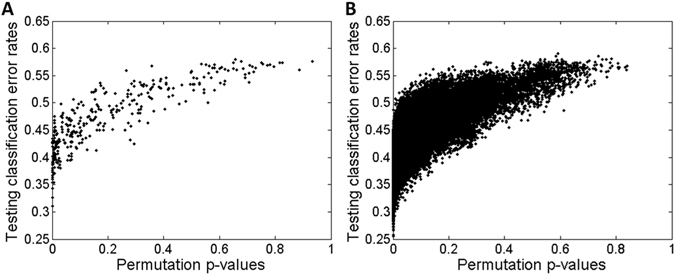
Necessity and feasibility of evaluating the performances of model-based perspective and boundary-based perspective. A 2D scattering plot denotes all the points with x-axis for p-values from permutations of the class label based on Welch’s t-test and y-axis for averages of classification error rates. Panel (A and B) correspond to the joint performances of each individual and pair of probes, respectively.

As has been declared, IHT was composed of not only model-based perspective but also boundary-based perspective^26^. Model-based perspective was equivalent to common hypothesis testing that developed a statistics to evaluate how different it could be between two populations of samples. As to boundary-based perspective, classifying each sample to the right phenotype group it belonged to could evaluate the performance using classification error rates by a formed hyperplane. In order to disclose the joint performances of IHT, we displayed a complementary nature of IHT. To each individual and each pair of probes, we made a permutation test based on Welch’s t-test after combined projection at step A1 (see Methods). P-values corresponding to each probe were obtained by 1 × 10^4^ random permutations of the class label (i.e., metastasis or non-metastasis). As to each pair of probes, a stepwise strategy was made in view of the larger amount of computation time for permutations on enumeration of each pair. We performed 1 × 10^4^ random permutations of the class label. Pairs with the smallest p-values (p = 0.0001) were selected for further 1 × 10^5^ random permutations. This procedure was repeated until the permutation times were 1 × 10^6^. Besides, Fisher’s LDA was also utilized as a classifier to each individual and each pair of probes (see Methods). Combined projection itself at step A1 formed a classifier on each probe. As to each pair, dimension reduction projection was provided. 1 × 10^4^ random re-sampling was made for cross-validation with 70% of the two groups (i.e., metastasis and non-metastasis) selected as a training set each time and the left samples as a testing set. The classification error rate was defined as an arithmetic mean between the two phenotype groups and calculated each time, considering that the sample size was not balanced between metastasis and metastasis-free group. As a result, an average of classification error rates from 1 × 10^4^ testing sets was obtained. Guided with the IHT thought, the average of classification error rates on testing sets and the corresponding p-value from the same individual and pair of probes formed a 2D scattering point. Together, 2D scattering points from all the enumerations formed a 2D scattering plot, as shown in Fig. 2.

From Fig. 2, we could see that model-based Welch’s p-values kept a different scale metric compared with boundary-based averages of classification error rates. If only using metric such as Euclidean distance, then averages of incorrect classification asserted the dominance. Put another way, individuals or pairs of probes with only small p-values would be submerged in those with small averages of classification error rates. In order to solve this problem, we sought to screen only on individuals or pairs with the top 10% p-values using bootstrapping each time. The rank of Euclidean distance after simultaneously normalizing p-values and averages of classification error rates was recorded each time. Then, averages of ranks and the corresponding standard deviations were obtained for selection of individuals or pairs of probes.

Anyway, interception of individuals or pairs with the top 10% p-values was too subjective. Thus, we considered to perform a bootstrap technique by selecting 90% samples in each round. We kept calculating the p-value and the average of classification error rates of each individual and pair of probes in each round. Then, we ranked individuals and pairs of probes by p-values and averages of classification error rates in an ascending order, respectively. Using the two orders, we voted for each individuals and pairs with a strategy as follows. Individuals or pairs with their rankings from No. 1 to No. 3 kept 20 scores. Those at the ranking from No. 4 to No. 5 obtained 15 scores. Those with their ranking from No. 6 to No. 10 kept ten scores. Those with their positions from No. 11 to No. 15 got five scores. Those gained one score with their rankings from No. 16 to No. 20. This strategy kept summing the scores from Welch’s p-value and the average of classification error rates of each round, when both of the two rankings were at the first 20. Otherwise, no score would be accumulated in this round. After 100 rounds of cycling step A2 and step A3, we obtained the accumulated scores of combinational rankings listed in Supplementary Tables S1 and S2 corresponding to individual feature and pair enumeration, respectively. On account of the computing time of pair enumeration, we made a broad screen using the same strategy on the whole samples, of which the ranking result could be seen in Supplementary Table S3.

### Affirmation of candidates at step A5 with their scores above threshold at step A4

At step A4, we chose those with their overall scores bigger than 200 for further analysis according to the strategy of assigning scores at step A3. In other words, individual or pair of probes gained at least two scores on average in each round should be chosen for further analysis. As a result, we selected 15 individuals of probes and 27 pairs of probes for further affirmation (see Supplementary Tables S1 and S2).

**Figure 3:**
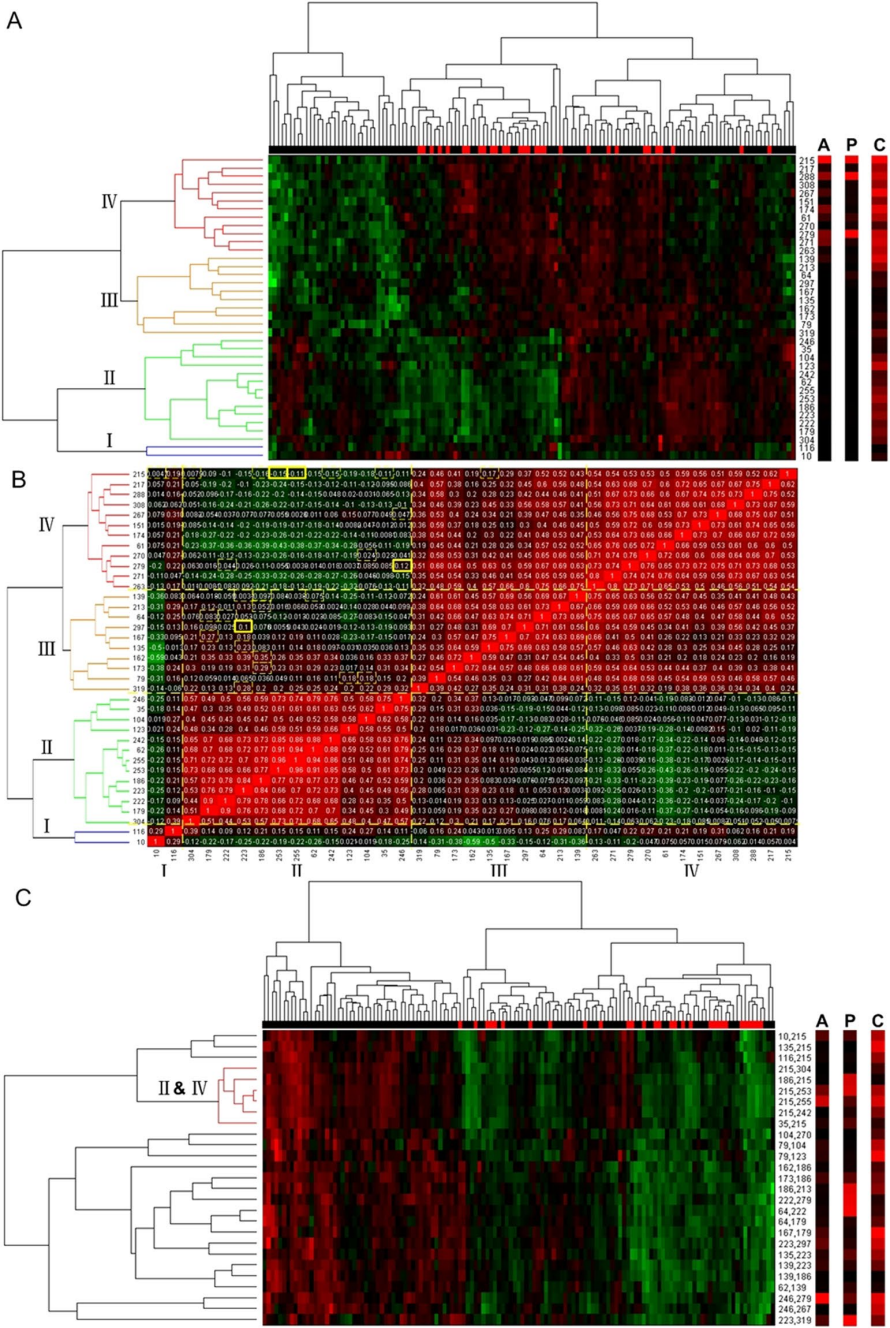
Correlation analyses at step A5. Row labels instead of probe names are illustrated in display convenience. The corresponding probe names can be found in Supplementary Tables S1 and S2. Bars labeled with A, P and C correspond to the overall scores of A5 accumulation, p-values and classification error rates, respectively. Panel (A) indicates a hierarchical clustering with complete linkage and centered Pearson correlation after z-score transformation of linear expression projections at step A1. Panel (B) contains the correlations reordered by the clustering results of Panel (A). Correlations of the 27 pairs chosen at step A4 are labeled with yellow boxes, of which the most significant pairs are labeled in bold. Panel (C) is also a hierarchical clustering under the same treatment as that of Panel (A), except that it is for each pair of probes.

We made correlation analyses at step A5. First, a union set of the 15 individuals and 27 pairs was obtained. A hierarchical clustering with complete linkage and centered Pearson correlation was made on combined projection values after z-score transformation for each element of the union set (see Fig. 3A). It could be seen in Fig. 3A that metastasis (red unit of horizonal bar) and metastasis-free samples (black unit of horizonal bar) were clear separated. Besides, probes were clustered into four groups, which might possibly correspond to four potential cliques. Second, we calculated correlations between each pair of the elements and reordered the probes by the clustering results of Fig. 3A (see Fig. 3B). Correlations of the 27 pairs of probes chosen at step A4 were labeled with yellow boxes. Third, we made another hierarchical clustering with complete linkage and centered Pearson correlation on dimension reduction projection values after z-score transformation for each pair of probes (see Fig. 3C). In the same way, it could be seen that metastasis samples (red unit of horizonal bar) were separated with only one misclassified. Besides, we discovered a cluster that right explained the relationship between group II and group IV.

According to the 27 pairs of probes chosen at step A4, we selected two pairs of miRNAs (i.e., miR-210 and miR-30c, miR-338 and miR-29b) that indicated the relationships between group II and group IV (see Fig. 3B and Supplementary Table S2). Besides, it was found that miRNAs in group IV mainly corresponded to the top significant individuals of probes at Supplementary Table S1 (also see Fig. 3A), which indicated that group IV might be a functional clique. Moreover, it could be seen in Fig. 3A that miRNAs in group IV were up-expressed, which indicated that the miRNAs in this potential clique could probably be prometastatic.

### Comparisons with other methods and independent validations

Many algorithms^31–35^ exist for selecting genes on expression profiles. In order to illustrate the effectiveness of our method, we selected recursive cluster elimination (RCE)^33^ and random forest (RF)^35^ for feature selection on dataset GSE6857 and made comparisons with our method. Following the steps of algorithm SVM_RCE (with its parameter, n = 100, m = 2, d = 0.1, r = 100, f = 0.3)^33^, we obtained 18 miRNA probes. After 1 × 10^4^ random re-sampling for cross-validation with 70% of the two groups (i.e., metastasis and non-metastasis) selected as a training set each time and the left samples as a testing set on combined projection, we got the average accuracies using SVM and Fisher’s LDA. Meanwhile, we made a permutation test at 1 × 10^4^ times based on Welch’s t-test after combined projection. The corresponding p-value was calculated. As to RF^35^, same evaluating indicators were calculated on 30 miRNA probes derived from RF, except that we changed the average accuracy of SVM to that of RF. As shown in Fig. 3A, the union set of the 15 individuals and 27 pairs selected using our method was simultaneously considered, and the same evaluating indicators were calculated. The selected miRNA probes derived from three comparative methods could be seen in Supplementary Table S4. Experimental results for comparison together with the p-values and the average accuracies of the selected two pairs of miRNAs considered to be signficant, were listed in Table 1.

**Table 1:** Comparisons among RCE^33^, RF^35^ and our method on dataset GSE6857.

Methods	Number of miRNAs or miRNA pairs	P-value	Accuracy using Fisher’s LDA	Accuracy using SVM	Accuracy using RF
RCE	18	0.0001	0.5332	0.5102	—
RF	30	0.0001	0.5141	—	0.5547
our method	37	0.0001	0.8117	—	—
our method	hsa-mir-29b-1No1	0.0001	0.7276	—	—
	hsa-mir-338No1				
our method	hsa-mir-30c-2No1	0.0001	0.7276	—	—
	hsa-mir-210-prec				
our method	hsa-mir-30c-1No1	0.0001	0.7161	—	—
	hsa-mir-210-prec				

In order to shown the effectiveness the selected pairs of miRNAs considered to be signaficant, we chose three datasets (i.e., GSE76903^36^, GSE67138 and GSE67139) for further independent validation. GSE76903 kept 20 patients with primary tumor, portal vein tumor thrombosis (PVTT) and adjacent normal. As to GSE67138 and GSE67139, 57 and 120 different patients with either only primary tumor or tumor vascular invasion were considered. Thus, we made a bilinear projection on GSE76903 and a dimension reduction projection on GSE76903, GSE67138 and GSE67139 using the selected pairs of miRNAs to be significant. After 1 × 10^4^ random re-sampling for cross-validation with 70% of the two groups (i.e., metastasis and non-metastasis) selected as a training set each time and the left samples as a testing set, we obtained the average accuracies using Fisher’s LDA. Besides, p-values corresponding to a permutation test at 1 × 10^4^ times based on Welch’s t-test were calculated. The experimental results were listed in Table 2.

**Table 2:** Independent validations on dataset GSE76903^36^, GSE67138 and GSE67139 using the selected miRNA pairs considered to be significnat.

Dataset	GSE76903	GSE76903	GSE67138	GSE67139
Sample size	40	40	57	120
Method	Bilinear projection	Dimension reduction projection	Dimension reduction projection	Dimension reduction projection
P-value using hsa-miR-29b and hsa-miR-338	0.8474	0.8528	0.0001	0.0001
Accuracy using hsa-miR-29b and hsa-miR-338	0.3942	0.3983	0.8106	0.8510
P-value using hsa-miR-30c and hsa-miR-210	0.3745	0.3678	0.0001	0.0001
Accuracy using hsa-miR-30c and hsa-miR-210	0.4810	0.4757	0.9340	0.6930

### Selection of potential targets regulated by miRNA candidates and KEGG pathway analysis

Once the significant pairs of miRNAs were selected, we had to deal with the matter how each two miRNAs were coordinated. A measure of miRNA interaction based on sequence and structure similarity^37^ was utilized and no similarity was measured between each selected pair. As a result, we focused on selection of potential target genes. One possible way was that they regulated the same target genes associated with HCC metastasis. The other way supposed that each one of the two miRNAs regulated different target genes, which together participated in a certain pathway. Based on the above two possibilities, we concentrated on selecting potential targets regulated by each miRNA pair and made futher KEGG pathway analysis.

First, we got potential target genes of each miRNA from the selected pairs using TarBase^28^, which provided miRNA/gene interactions with high quality experimental validations. Second, intersections and unions of target genes from each selected miRNA pair were made. Third, we applied DAVID^29^ to obtain KEGG pathways corresponding to targets from not only single miRNA of each selected pair but also the intersection and union in each miRNA pair. Results in detail were in Supplementary Tables S5 and S6. Last but foremost, we listed the most significant pathways in Tables 3 and 4. Considering either of the two significant miRNA pairs consisted of one miRNA from group IV and the other from group II (see Fig. 3B), we concluded from Tables 3 and 4 that p53 signaling pathway could probably be the common pathway regulated by significant miRNA pairs associated with HCC venous metastasis. Table 5 illustrated the potential target genes in p53 signaling pathway, with common genes targeted by both two significant pairs labeled in bold.

**Table 3:** Significant KEGG pathways corresponding to target union from miR-210 and miR-30c, with all of the Bonferroni, Benjamini and FDR values smaller than 0.05.

Term	P-value	Bonferroni	Benjamini	FDR
hsa04120:Ubiquitin mediated proteolysis	2.91 × 10^−11^	7.85 × 10^−9^	7.85 × 10^−9^	3.82 × 10^−8^
hsa04115:p53 signaling pathway*	3.99 × 10^−6^	1.08 × 10^−3^	5.39 × 10^−4^	5.25 × 10^−3^
hsa05200:Pathways in cancer	1.19 × 10^−5^	3.21 × 10^−3^	1.07 × 10^−3^	1.57 × 10^−2^
hsa04114:Oocyte meiosis	2.72 × 10^−5^	7.33 × 10^−3^	1.84 × 10^−3^	3.58 × 10^−2^
hsa04141:Protein processing in endoplasmic reticulum*	3.54 × 10^−5^	9.51 × 10^−3^	1.91 × 10^−3^	4.65 × 10^−2^

*Bold pathways correspond to significant pathways which are insignificant using targets from either miR-210 or miR-30c.

**Table 4:** Significant KEGG pathways corresponding to target union from miR-338 and miR-29b, with all of the Bonferroni, Benjamini and FDR values smaller than 0.05.

Term	P-value	Bonferroni	Benjamini	FDR
hsa04510:Focal adhesion	2.51 × 10^−9^	6.85 × 10^−7^	6.85 × 10^−7^	3.30 × 10^−6^
hsa04110:Cell cycle	1.60 × 10^−7^	4.36 × 10^−5^	2.18 × 10^−5^	2.10 × 10^−4^
hsa05210:Colorectal cancer	3.28 × 10^−7^	8.95 × 10^−5^	2.98 × 10^−5^	4.32 × 10^−4^
hsa04151:PI3K-Akt signaling pathway	7.05 × 10^−7^	1.92 × 10^−4^	4.81 × 10^−5^	9.28 × 10^−4^
hsa05161:Hepatitis B	5.94 × 10^−6^	1.62 × 10^−3^	3.24 × 10^−4^	7.83 × 10^−3^
hsa05200:Pathways in cancer	6.41 × 10^−6^	1.75 × 10^−3^	2.92 × 10^−4^	8.45 × 10^−3^
hsa05166:HTLV-I infection	1.38 × 10^−5^	3.76 × 10^−3^	5.37 × 10^−4^	1.82 × 10^−2^
hsa05222:Small cell lung cancer	1.96 × 10^−5^	5.34 × 10^−3^	6.69 × 10^−4^	2.58 × 10^−2^
hsa04115:p53 signaling pathway*	2.27 × 10^−5^	6.17 × 10^−3^	6.87 × 10^−4^	2.98 × 10^−2^
hsa05215:Prostate cancer	3.44 × 10^−5^	9.35 × 10^−3^	9.39 × 10^−4^	4.53 × 10^−2^

*Bold pathway corresponds to significant pathway which is insignificant using targets from either miR-338 or miR-29b.

**Table 5:** Genes targeted by significant miRNA pairs in p53 signaling pathway.

KEGG pathway	Genes targeted by either miR-210 or miR-30c				Genes targeted by either miR-338 or miR-29c			
p53 signaling pathway	STEAP3	CDK1	CDK6*	RRM2B	CYCS	TP53	CDK6*	SESN2
	PMAIP1	CCNG1	SESN3	CCNB1*	SESN1	PTEN	GTSE1	ATM
	CCNE2	CASP3	CCND1*	CCND2*	CCNB1*	CCNE1	PPM1D	CDKN1A
	RRM2	SERPINE1	DDB2	MDM2*	CCND1*	CCND2*	BAX	CASP8
	SIAH1*	FAS	THBS1*	IGFBP3	MDM2*	SIAH1*	THBS1*	

*Bold genes represent the common genes targeted by both two significant miRNA pairs.

## Discussion

Using joint covariate detection, we identified two new miRNA pairs (i.e., miR-210 and miR-30c, miR-338 and miR-29b) associated with venous metastasis in primary HCC. Among them, miR-210^9^ and miR-29b^5^ have been explicitly reported to be prometastatic or antimetastatic. Main contributions were listed as follows.

First, we practically combined tumor and adjacent non-tumor expressions together based on Fisher’s LDA. Inevitably, useful information was discarded when tumor and adjacent non-tumor tissues were treated separately. In addition, adjacent non-tumor tissues were always regarded as background, and that led to an inappropriate subtraction between the tumor log2 expressions and the corresponding background. In fact, this only provided a linear combination using a special linear projection on the counter-diagonal orientation with tumor and adjacent normal expressions to be coordinates. Instead, Fisher’s LDA-based combination was utilized, which corresponded to an optimal combination with a best discriminative performance.

Second, we enumerated miRNA pairs in practice, on account of their potential cooperative functions. There used to be a research that added or subtracted the log2 expressions from two individually selected miRNAs as a better discriminative performance having been reported^20^. However, it was still a linear projection between the expressions from two different miRNAs on diagonal or counter-diagonal orientation. In order to solve the problems mentioned above, a matrix-variate hypothesis testing^26^ that considered tumor and adjacent non-tumor expressions from two different miRNAs was introduced in. Essentially, we simplified bilinear projection as a two-step linear projection (see Fig. 1B) based on Fisher’s LDA due to the limited sample size that led to poor covariance matrixs, and fulfilled enumerations of miRNA pairs. As to single miRNAs, the matrix-variate hypothesis testing was simplified into a multivariate hypothesis testing on tumor and adjacent non-tumor expressions of each sample group. Enumeration on miRNA pairs instead of only individual miRNAs which were thought to be significant indicated the superiority, after a comparison on affirmation with hierarchical clustering was made between Fig. 3C and Supplementary Fig. S1. It could be seen in Supplementary Fig. S1 that metastasis samples (red unit of horizonal bar) were separated with six misclassified, when only individual miRNAs which were thought to be significant were considered.

Third, we simultaneously integrated class comparison with class prediction instead of the ordinal way that regarded class prediction only as a posterior validation of selected miRNAs. In fact, this viewpoint derived from IHT^30^. Class comparison corresponded to distribution-based hypothesis testing that aimed at evaluating whether two populations of samples were significantly different. As to class prediction, it was to classify samples into their corresponding populations by a discriminant rule, and was named as supervised classification. The joint performances of IHT displayed a complementary nature between class comparison and class prediction. In other words, miRNAs or pairs with good discriminative performances should not only have big differences between two populations but also keep small classification error rates meanwhile. In fact, the superiority of using IHT could be seen in Fig. 3C. Compared with vertical bars P and C, vertical bar A was more accurate and could overcome the selection of false positive features.

Fourth, an A5 formulation^30^ was emulated in order to overcome the problem of small sample size compared with large feature numbers. Voting strategy was hierarchically designed for better selection of individual miRNAs and pairs. Besides, hierarchical clustering and correlation analysis were made on candidates to identify significant miRNA pairs, as shown in Fig. 3. In the end, potential targets were selected using TarBase^28^, and a KEGG pathway probably associated with venous metastasis in primary HCC was selected using DAVID^29^.

As shown in Fig. 3A, miRNA combinations obtained from step A1 to step A4 could be used to distinguish metastasis samples with non-metastasis ones. In Fig. 3A, the black units in the metastasis part of the horizonal bar probably indicated potential metastasis. Besides, these miRNAs assembled in four groups, which probably corresponded to four potential cliques. In addition, Fig. 3B illustrated that the components of each significant miRNA pair derived from different miRNA cliques. It could also be seen in Fig. 3C that the significant miRNA pairs assembled in a cluster derived from group II and group IV, which might indicated that each significant miRNA pair was composed of one miRNA from individually significant group (i.e., group IV) and the other from insignificant group (i.e., group II). The function of each miRNA clique is still under discussion. However, the phenomena mentioned above may help in further enumeration of higher feature tuples.

From Table 1 we could see that none of the three methods (i.e., RCE^33^, RF^35^ and our method) perform perfect. Actually, this phenomenon derives from the existence of probably potential metastasis in metastasis-free group on dataset GSE6857. Anyway, the average accuracy of our method using Fisher’s LDA is better than that of RCE and RF, especially that the performance of the selected miRNA pairs considered to be significant using our enumeration method is better than that of the miRNA groups derived from RCE and RF. This phenomenon further emphasize that the components of each significant miRNA pair may derive from different miRNA cliques. As shown in Table 2, independent validations on dataset GSE67138 and GSE67139 work well, which demonstrates the significance of the two selected miRNA pairs. Anyway, either bilinear projection or dimension reduction projection perform poor using dataset GSE76903, which may indicate a temporal correlation among spatial tissues of the same HCC patients (i.e., primary tumor, portal vein tumor thrombosis and adjacent normal).

Significantly different from pathways associated with each component of a significant miRNA pair, two pathways in bold were achieved in Tables 3 and 4. On assumption that the selected two significant miRNA pairs kept the same function, p53 signaling pathway commonly existed in Tables 3 and 4 was chosen for further analysis. As shown in Table 5, there were seven common genes in p53 signaling pathway among the targets of the two significant miRNA pairs, three of which were retrieved on Web of Science to be directly associated with metastasis in HCC. CDK6^37^ was thought to participate in migration of HCC, although it was dispensable for p16-enhanced migration. CCND1^38^ was regarded to account for cell proliferation, recurrence and metastasis in HCC. Besides, functional association of MDM2^39^ binding protein with metastatic potential of hepatocellular carcinoma was found. All these literatures indirectly supported that parts of p53 signaling pathway (especially the common target genes) participate in HCC venous metastasis, which might provide the evidences of identified miRNA pairs and further certificate the effectiveness of our method.

## Methods

### Fisher’s linear projection and classification

We utilized Fisher’s LDA to fulfill combined projection between HCC and adjacent normal tissues and accomplish dimension reduction projection within each miRNA pair. The orientation from Fisher’s linear projection was used to separate the projected samples. Weight vector **w** with the most discriminative performance was expressed as,

1 $${\bf{w}}={{\bf{S}}}_{w}^{-1}({{\bf{m}}}_{1}-{{\bf{m}}}_{2}),$$

where **m**_1_ and **m**_2_ were the 2-dimensional sample mean of HCC and adjacent non-tumor tissues for combined projection or the 2-dimensional sample mean of column vectors representing combined projection values on a miRNA pair for dimension reduction projection, respectively. **S**_*w*_ was called the within-class scatter matrix, and was given by,

2 $${{\bf{S}}}_{w}=\frac{{n}_{1}{{\sum }_{{\bf{x}}\in {D}_{1}}({\bf{x}}-{{\bf{m}}}_{1})({\bf{x}}-{{\bf{m}}}_{1})}^{t}+{n}_{2}{\sum }_{{\bf{x}}\in {D}_{2}}({\bf{x}}-{{\bf{m}}}_{2})({\bf{x}}-{{\bf{m}}}_{2}{)}^{t}}{{n}_{1}+{n}_{2}},$$

where **x** denoted a 2-dimensional sample from HCC or adjacent non-tumor tissues for combined projection or a 2-dimensional sample from combined projection values for dimension reduction projection. *n*_1_ and *n*_2_ were the numbers of samples that represented metastasis-free and metastasis samples with *n*_1_ = 102 and *n*_2_ = 29. Detail formula derivation could be seen in Duda’s book^25^. The Fisher’s LDA-based classification was based on the optimal decision boundary with its equation **w** ^*t*^ **x** + **w**_0_ = 0, where **w**_0_ was given by ${{\bf{w}}}_{0}={\bf{w}}({{\bf{m}}}_{1}+{{\bf{m}}}_{2})/2$. **x** was regarded as a non-metastasis sample when **w** ^*t*^ **x** + **w**_0_ < 0, and vice versa. Therefore, the error rate that wrongly classified **x** into the non-metastasis group was defined as $E{r}_{1}=\#\{{{\bf{w}}}^{t}{\bf{x}}+{{\bf{w}}}_{0} > =0\}/{n}_{1}$, and the error rate that wrongly classified **x** into the metastasis group was $E{r}_{2}=\#\{{{\bf{w}}}^{t}{\bf{x}}+{{\bf{w}}}_{0} < 0\}/{n}_{2}$. Considering that the sample size was not balanced between the two phenotype groups, the classification error rate was defined as *Er* = (*Er*_1_ + *Er*_2_)/2.

### A permutation test based on univariate Welch’s t-test

We tested combined projection values of each individual of probes or dimension reduction projection values of each pair of probes using Welch’s t-test, which was expressed as,

3 $$t(\upsilon (i))=\frac{{m}_{2}(i)-{m}_{1}(i)}{\sqrt{\tfrac{{s}_{1}^{2}(i)}{{n}_{1}}+\tfrac{{s}_{2}^{2}(i)}{{n}_{2}}}},$$

where *m*_1_(*i*), ${s}_{1}^{2}(i)$, *m*_2_(*i*) and ${s}_{2}^{2}(i)$ were the sample mean and variance of the metastasis-free and metastasis group according to the *i*-th probe or probe pair from enumeration. The *i*-th freedom degree was defined as $\upsilon (i)=\tfrac{{({s}_{1}^{2}(i)/{n}_{1}+{s}_{2}^{2}(i)/{n}_{2})}^{2}}{{s}_{1}^{4}(i)/[{n}_{1}^{2}\cdot ({n}_{1}-\mathrm{1)]}+{s}_{2}^{4}(i)/[{n}_{2}^{2}\cdot ({n}_{2}-\mathrm{1)]}}$. Corresponding p-value was obtained by inspecting the t-distribution table. In order to enlarge sample size, we considered to use a permutation method. Under the assumption that there were no differential expressions between metastasis and metastasis-free group, the *i*-th t statistics obeyed the same distribution regardless of how we made the assignments of group labels. Therefore, the p-value for the *i*-th statistic was calculated by,

4 $$p(i)=\sum _{b=1}^{B}\,\frac{\#\{|{t}_{0}(i)|\ge |t(i)|\}}{B},$$

where *t*_0_ represented a null statistics by a random rearrangement of class label with *B* to be the times of permutation.

### IHT, voting and accumulation

In each round of re-sampling, IHT combined the error rate *Er* based on boundary perspective with p-value derived from model perspective. Considering different scales about the spatial distribution of scatters (p-value, *Er*) representing individuals or pairs of probes, a voting strategy was made. In other words, individuals or pairs of probes were ranked by p-values and averages of *Er*s in an ascending order, respectively. Votes from the two aspects were accumulated together in order to select candidate individual miRNAs or pairs. In detail, the voting strategy was proposed as Table 6.

**Table 6:** Voting strategy at step A3.

Rank	Votes in each round
No.1–No.3	20
No.4–No.5	15
No.6–No.10	10
No.11–No.15	5
No.16–No.20	1
Otherwise	0

## Electronic supplementary material

Supplementary materials
