# Supplementary material for: Joint Covariate Detection on Expression Profiles for Identifying MicroRNAs Related to Venous Metastasis in Hepatocellular Carcinoma
Source: Sci Rep. 2017 Jul 13;7:5349. doi: 10.1038/s41598-017-05776-1 (PMC5509738; doi:10.1038/s41598-017-05776-1)
Supplement: Supplementary file 1 — Supplementary materials [file 41598_2017_5776_MOESM1_ESM.rtf]

Joint Covariate Detection on Expression Profiles for Identifying MicroRNAs Related to Venous Metastasis in Hepatocellular Carcinoma 

Xudong Zhao1 , Lei Wang1 and Guangsheng Chen1,*

1Northeast Forestry University, College of Information and Computer Engineering, Harbin, 150001, China
*chengs.nefu@gmail.com


Supplementary materials including:
Supplementary Table S1;
Supplementary Table S2;
Supplementary Table S3;
Supplementary Table S4;
Supplementary Table S5;
Supplementary Table S6;

Supplementary Figure S1.


Supplementary Table S1. Accumulated scores with individual probes on 90% samples
miRNA probe	Row numbe	r	Overall scores of A5 accumulation	
hsa-mir-210-prec	215	3775	
hsa-mir-346No1	288	2230	
hsa-mir-338No1	279	1995	
hsa-mir-185-precNo2	174	1945	
hsa-mir-326No2	271	1910	
hsa-mir-324No2	267	1285	
hsa-mir-030b-precNo2	61	1020	
hsa-mir-138-2-prec	139	955	
hsa-mir-212-precNo1	217	690	
hsa-mir-323No2	263	415	
hsa-mir-3p21-v1/v2-sense5P	308	315	
hsa-mir-1-2No1	123	290	
hsa-mir-30c-1No1	253	265	
hsa-mir-149-prec	151	260	
hsa-mir-215-precNo2	223	210	
hsa-mir-3p21-v3/v4-sense25P	316	170	
hsa-mir-128a-precNo1	115	160	
hsa-mir-206-precNo2	213	125	
hsa-mir-335No1	276	110	
hsa-mir-194-precNo1	186	105	
hsa-mir-124a-2-prec	104	90	
hsa-mir-211-precNo2	216	60	
hsa-mir-374No1	304	55	
hsa-mir-206-precNo1	212	50	
hsa-mir-30eNo1	257	45	
hsa-mir-007-2-precNo2	21	40	
hsa-mir-345No2	287	40	
hsa-mir-192-2/3No1	179	35	
hsa-mir-028-prec	54	30	
hsa-mir-192No1	181	30	
hsa-mir-148aNo1	147	25	
hsa-mir-30c-2No1	255	25	
hsa-mir-3p21-v3/v4-sense45P	319	25	
hsa-mir-126No1	111	20	
hsa-mir-016a-chr13	33	15	
hsa-mir-328No1	272	15	
hsa-mir-030c-prec	62	10	
hsa-mir-194-2No1	185	10	
hsa-mir-196a-2No1	193	10	
hsa-let-7a-1-prec	1	0	
hsa-let-7a-2-precNo1	2	0	
hsa-let-7a-2-precNo2	3	0	
hsa-let-7a-3-prec	4	0	
hsa-let-7b-prec	5	0	


hsa-let-7c-prec	6	0	
hsa-let-7d-prec	7	0	
hsa-let-7d-v1-prec	8	0	
hsa-let-7d-v2-precNo1	9	0	
hsa-let-7d-v2-precNo2	10	0	
hsa-let-7e-prec	11	0	
hsa-let-7f-1-precNo1	12	0	
hsa-let-7f-1-precNo2	13	0	
hsa-let-7f-2-prec2	14	0	
hsa-let-7g-precNo1	15	0	
hsa-let-7g-precNo2	16	0	
hsa-let-7iNo1	17	0	
hsa-let-7iNo2	18	0	
hsa-mir-001b-1-prec1	19	0	
hsa-mir-007-1-prec	20	0	
hsa-mir-007-3-precNo1	22	0	
hsa-mir-007-3-precNo2	23	0	
hsa-mir-009-1No1	24	0	
hsa-mir-009-3No1	25	0	
hsa-mir-009-3No2	26	0	
hsa-mir-010a-precNo1	27	0	
hsa-mir-010b-precNo1	28	0	
hsa-mir-010b-precNo2	29	0	
hsa-mir-015a-2-precNo1	30	0	
hsa-mir-015a-2-precNo2	31	0	
hsa-mir-015b-precNo1	32	0	
hsa-mir-016b-chr3	34	0	
hsa-mir-017-precNo1	35	0	
hsa-mir-017-precNo2	36	0	
hsa-mir-019a-prec	37	0	
hsa-mir-019b-1-prec	38	0	
hsa-mir-019b-2-prec	39	0	
hsa-mir-020-prec	40	0	
hsa-mir-021-prec-17No1	41	0	
hsa-mir-022-prec	42	0	
hsa-mir-023a-prec	43	0	
hsa-mir-023b-prec	44	0	
hsa-mir-024-1-precNo1	45	0	
hsa-mir-024-1-precNo2	46	0	
hsa-mir-024-2-prec	47	0	
hsa-mir-025-prec	48	0	
hsa-mir-026a-precNo1	49	0	
hsa-mir-026a-precNo2	50	0	
hsa-mir-026b-prec	51	0	
hsa-mir-027a-prec	52	0	
hsa-mir-027b-prec	53	0	


hsa-mir-029a-2No1	55	0	
hsa-mir-029a-2No2	56	0	
hsa-mir-029c-prec	57	0	
hsa-mir-030a-precNo1	58	0	
hsa-mir-030a-precNo2	59	0	
hsa-mir-030b-precNo1	60	0	
hsa-mir-030d-precNo2	63	0	
hsa-mir-031-prec	64	0	
hsa-mir-032-precNo1	65	0	
hsa-mir-032-precNo2	66	0	
hsa-mir-033b-prec	67	0	
hsa-mir-033-prec	68	0	
hsa-mir-034precNo1	69	0	
hsa-mir-034-precNo2	70	0	
hsa-mir-092-prec-13=092-1No1	71	0	
hsa-mir-092-prec-13=092-1No2	72	0	
hsa-mir-092-prec-X=092-2	73	0	
hsa-mir-093-prec-7.1=093-1	74	0	
hsa-mir-095-prec-4	75	0	
hsa-mir-096-prec-7No1	76	0	
hsa-mir-096-prec-7No2	77	0	
hsa-mir-098-prec-X	78	0	
hsa-mir-099b-prec-19No1	79	0	
hsa-mir-099b-prec-19No2	80	0	
hsa-mir-099-prec-21	81	0	
hsa-mir-100-1/2-prec	82	0	
hsa-mir-100No1	83	0	
hsa-mir-101-1/2-precNo1	84	0	
hsa-mir-101-1/2-precNo2	85	0	
hsa-mir-101-1No2	86	0	
hsa-mir-101-prec-9	87	0	
hsa-mir-102-prec-1	88	0	
hsa-mir-103-2-prec	89	0	
hsa-mir-103-prec-5=103-1	90	0	
hsa-mir-105-2No1	91	0	
hsa-mir-105-prec-X.1=105-1	92	0	
hsa-mir-106aNo1	93	0	
hsa-mir-106bNo1	94	0	
hsa-mir-106bNo2	95	0	
hsa-mir-106-prec-X	96	0	
hsa-mir-107No1	97	0	
hsa-mir-107-prec-10	98	0	
hsa-mir-1-1No2	99	0	
hsa-mir-122a-prec	100	0	
hsa-mir-123-precNo1	101	0	
hsa-mir-123-precNo2	102	0	


hsa-mir-124a-1-prec1	103	0	
hsa-mir-124a-3-prec	105	0	
hsa-mir-125a-precNo1	106	0	
hsa-mir-125a-precNo2	107	0	
hsa-mir-125b-1	108	0	
hsa-mir-125b-2-precNo2	109	0	
hsa-miR-126No1	110	0	
hsa-mir-126No2	112	0	
hsa-miR-126No2	113	0	
hsa-mir-127-prec	114	0	
hsa-mir-128a-precNo2	116	0	
hsa-mir-128b-precNo1	117	0	
hsa-mir-128b-precNo2	118	0	
hsa-mir-129-2No1	119	0	
hsa-mir-129-2No2	120	0	
hsa-mir-129-precNo1	121	0	
hsa-mir-129-precNo2	122	0	
hsa-mir-1-2No2	124	0	
hsa-mir-130a-precNo2	125	0	
hsa-mir-130bNo1	126	0	
hsa-mir-130bNo2	127	0	
hsa-mir-132-precNo1	128	0	
hsa-mir-132-precNo2	129	0	
hsa-mir-133a-1	130	0	
hsa-mir-133bNo2	131	0	
hsa-mir-135-1-prec	132	0	
hsa-mir-135-2-prec	133	0	
hsa-mir-135a-1No1	134	0	
hsa-mir-135a-2No1	135	0	
hsa-mir-135bNo1	136	0	
hsa-mir-136-precNo2	137	0	
hsa-mir-138-1-prec	138	0	
hsa-mir-139-prec	140	0	
hsa-mir-140No2	141	0	
hsa-mir-142-prec	142	0	
hsa-mir-143-prec	143	0	
hsa-mir-145-prec	144	0	
hsa-mir-146-prec	145	0	
hsa-mir-147-prec	146	0	
hsa-mir-148bNo1	148	0	
hsa-mir-148bNo2	149	0	
hsa-mir-148-prec	150	0	
hsa-mir-150-prec	152	0	
hsa-mir-151-prec	153	0	
hsa-mir-152-precNo1	154	0	
hsa-mir-152-precNo2	155	0	


hsa-mir-153-1-prec1	156	0	
hsa-mir-155-prec	157	0	
hsa-mir-15aNo1	158	0	
hsa-mir-16-1No1	159	0	
hsa-mir-16-2No1	160	0	
hsa-mir-16-2No2	161	0	
hsa-mir-181a-precNo1	162	0	
hsa-mir-181a-precNo2	163	0	
hsa-mir-181b-1No1	164	0	
hsa-mir-181b-1No2	165	0	
hsa-mir-181b-2No1	166	0	
hsa-mir-181b-2No2	167	0	
hsa-mir-181b-precNo1	168	0	
hsa-mir-181c-precNo1	169	0	
hsa-mir-181c-precNo2	170	0	
hsa-mir-182-precNo1	171	0	
hsa-mir-184-precNo2	172	0	
hsa-mir-185-precNo1	173	0	
hsa-mir-186-prec	175	0	
hsa-mir-187-precNo1	176	0	
hsa-mir-188-prec	177	0	
hsa-mir-191-prec	178	0	
hsa-mir-192-2/3No2	180	0	
hsa-mir-193-precNo1	182	0	
hsa-mir-193-precNo2	183	0	
hsa-mir-194-1No1	184	0	
hsa-mir-195-prec	187	0	
hsa-mir-196-1-precNo1	188	0	
hsa-mir-196-1-precNo2	189	0	
hsa-mir-196-2-precNo2	190	0	
hsa-mir-196a-1No1	191	0	
hsa-mir-196a-1No2	192	0	
hsa-mir-196bNo1	194	0	
hsa-mir-196bNo2	195	0	
hsa-mir-197-prec	196	0	
hsa-mir-198-prec	197	0	
hsa-mir-199a-1-prec	198	0	
hsa-mir-199a-2-prec	199	0	
hsa-mir-199b-precNo1	200	0	
hsa-mir-199b-precNo2	201	0	
hsa-mir-200a-prec	202	0	
hsa-mir-200bNo1	203	0	
hsa-mir-200bNo2	204	0	
hsa-mir-200cNo1	205	0	
hsa-mir-200cNo2	206	0	
hsa-mir-202-prec	207	0	


hsa-mir-203-precNo1	208	0	
hsa-mir-204-precNo1	209	0	
hsa-mir-204-precNo2	210	0	
hsa-mir-205-prec	211	0	
hsa-mir-208-prec	214	0	
hsa-mir-212-precNo2	218	0	
hsa-mir-213-precNo1	219	0	
hsa-mir-213-precNo2	220	0	
hsa-mir-214-prec	221	0	
hsa-mir-215-precNo1	222	0	
hsa-mir-216-precNo1	224	0	
hsa-mir-218-1-prec	225	0	
hsa-mir-218-2-precNo1	226	0	
hsa-mir-218-2-precNo2	227	0	
hsa-mir-219-1No1	228	0	
hsa-mir-219-1No2	229	0	
hsa-mir-219-2No1	230	0	
hsa-mir-219-2No2	231	0	
hsa-mir-219-prec	232	0	
hsa-mir-21No1	233	0	
hsa-mir-220-prec	234	0	
hsa-mir-221-prec	235	0	
hsa-mir-222-precNo1	236	0	
hsa-mir-222-precNo2	237	0	
hsa-mir-223-prec	238	0	
hsa-mir-224-prec	239	0	
hsa-mir-26a-1No1	240	0	
hsa-mir-26a-1No2	241	0	
hsa-mir-26a-2No1	242	0	
hsa-mir-26a-2No2	243	0	
hsa-mir-296No1	244	0	
hsa-mir-299No1	245	0	
hsa-mir-29b-1No1	246	0	
hsa-mir-29b-1No2	247	0	
hsa-mir-29b-2=102prec7.1=7.2	248	0	
hsa-mir-301No2	249	0	
hsa-mir-302aNo1	250	0	
hsa-mir-302aNo2	251	0	
hsa-mir-302bNo2	252	0	
hsa-mir-30c-1No2	254	0	
hsa-mir-30c-2No2	256	0	
hsa-mir-30eNo2	258	0	
hsa-mir-320No1	259	0	
hsa-mir-320No2	260	0	
hsa-mir-321No1	261	0	
hsa-mir-321No2	262	0	


hsa-miR-324-5pNo1	264	0	
hsa-miR-324-5pNo2	265	0	
hsa-mir-324No1	266	0	
hsa-mir-325No1	268	0	
hsa-mir-325No2	269	0	
hsa-mir-326No1	270	0	
hsa-mir-330No1	273	0	
hsa-mir-330No2	274	0	
hsa-mir-331No2	275	0	
hsa-mir-335No2	277	0	
hsa-mir-337No1	278	0	
hsa-mir-338No2	280	0	
hsa-mir-339No1	281	0	
hsa-mir-339No2	282	0	
hsa-mir-340No2	283	0	
hsa-mir-342No1	284	0	
hsa-mir-342No2	285	0	
hsa-mir-345No1	286	0	
hsa-mir-34aNo1	289	0	
hsa-mir-34bNo1	290	0	
hsa-mir-34bNo2	291	0	
hsa-mir-34cNo2	292	0	
hsa-mir-367No2	293	0	
hsa-mir-368No1	294	0	
hsa-mir-369No1	295	0	
hsa-mir-370No2	296	0	
hsa-mir-371No1	297	0	
hsa-mir-371No2	298	0	
hsa-mir-372No1	299	0	
hsa-mir-373No1	300	0	
hsa-miR-373No1	301	0	
hsa-mir-373No2	302	0	
hsa-miR-373No2	303	0	
hsa-mir-3p21-v1/v2-AntiS3P	305	0	
hsa-mir-3p21-v1/v2-AntiS5P	306	0	
hsa-mir-3p21-v1/v2-sense3P	307	0	
hsa-mir-3p21-v3/v4-AntiS13P	309	0	
hsa-mir-3p21-v3/v4-AntiS23P	310	0	
hsa-mir-3p21-v3/v4-AntiS33P	311	0	
hsa-mir-3p21-v3/v4-AntiS43P	312	0	
hsa-mir-3p21-v3/v4-sense13P	313	0	
hsa-mir-3p21-v3/v4-sense15P	314	0	
hsa-mir-3p21-v3/v4-sense23P	315	0	
hsa-mir-3p21-v3/v4-sense33P	317	0	
hsa-mir-3p21-v3/v4-sense35P	318	0	

Supplementary Table S2.  Accumulated scores with pair of probes on 90% samples

miRNA probe	
miRNA probe	
Row number	
Row number	Overall scores of
A5 accumulation	
hsa-mir-29b-1No1	hsa-mir-338No1	246	279	2283	
hsa-mir-210-prec	hsa-mir-30c-2No1	215	255	1892	
hsa-mir-210-prec	hsa-mir-30c-1No1	215	253	1092	
hsa-mir-215-precNo2	hsa-mir-371No1	223	297	1011	
hsa-let-7d-v2-precNo2	hsa-mir-210-prec	10	215	901	
hsa-mir-215-precNo2	hsa-mir-3p21-v3/v4-sense4	5	223	319	855	
hsa-mir-181b-2No2	hsa-mir-192-2/3No1	167	179	834	
hsa-mir-185-precNo1	hsa-mir-194-precNo1	173	186	812	
hsa-mir-138-2-prec	hsa-mir-215-precNo2	139	223	706	
hsa-mir-099b-prec-19No	hsa-mir-124a-2-prec	79	104	669	
hsa-mir-099b-prec-19No	hsa-mir-1-2No1	79	123	612	
hsa-mir-135a-2No1	hsa-mir-215-precNo2	135	223	604	
hsa-mir-017-precNo1	hsa-mir-210-prec	35	215	587	
hsa-mir-031-prec	hsa-mir-192-2/3No1	64	179	503	
hsa-mir-031-prec	hsa-mir-215-precNo1	64	222	401	
hsa-mir-194-precNo1	hsa-mir-206-precNo2	186	213	377	
hsa-mir-128a-precNo2	hsa-mir-210-prec	116	215	373	
hsa-mir-181a-precNo1	hsa-mir-194-precNo1	162	186	352	
hsa-mir-215-precNo1	hsa-mir-338No1	222	279	345	
hsa-mir-135a-2No1	hsa-mir-210-prec	135	215	330	
hsa-mir-030c-prec	hsa-mir-138-2-prec	62	139	326	
hsa-mir-194-precNo1	hsa-mir-210-prec	186	215	314	
hsa-mir-138-2-prec	hsa-mir-194-precNo1	139	186	306	
hsa-mir-29b-1No1	hsa-mir-324No2	246	267	286	
hsa-mir-210-prec	hsa-mir-26a-2No1	215	242	283	
hsa-mir-210-prec	hsa-mir-374No1	215	304	233	
hsa-mir-124a-2-prec	hsa-mir-326No1	104	270	217	
hsa-mir-030b-precNo1	hsa-mir-210-prec	60	215	178	
hsa-mir-210-prec	hsa-mir-3p21-v3/v4-sense4	215	319	152	
hsa-let-7d-v2-precNo2	hsa-mir-211-precNo2	10	216	138	
hsa-mir-192-2/3No2	hsa-mir-210-prec	180	215	131	
hsa-mir-185-precNo2	hsa-mir-372No1	174	299	119	
hsa-mir-210-prec	hsa-mir-338No1	215	279	114	
hsa-mir-030c-prec	hsa-mir-210-prec	62	215	103	
hsa-let-7g-precNo1	hsa-mir-210-prec	15	215	79	
hsa-mir-206-precNo2	hsa-mir-215-precNo2	213	223	75	
hsa-mir-196-1-precNo2	hsa-mir-210-prec	189	215	72	
hsa-mir-148-prec	hsa-mir-210-prec	150	215	72	
hsa-mir-181b-2No2	hsa-mir-192No1	167	181	57	
hsa-mir-019b-2-prec	hsa-mir-210-prec	39	215	51	
hsa-mir-210-prec	hsa-mir-215-precNo2	215	223	50	
hsa-mir-027a-prec	hsa-mir-210-prec	52	215	47	
hsa-mir-195-prec	hsa-mir-210-prec	187	215	42	


hsa-mir-210-prec	hsa-mir-335No1	215	276	40	
hsa-mir-192-2/3No1	hsa-mir-338No1	179	279	38	
hsa-mir-206-precNo2	hsa-mir-29b-2=102prec7.1=	7	213	248	33	
hsa-mir-1-2No1	hsa-mir-210-prec	123	215	32	
hsa-mir-030d-precNo2	hsa-mir-210-prec	63	215	18	
hsa-mir-148aNo1	hsa-mir-210-prec	147	215	17	
hsa-mir-105-2No1	hsa-mir-181a-precNo1	91	162	0	

Supplementary Table S3.  Accumulated scores with pair of probes on whole samples


miRNA probe	

miRNA probe	

Row	

Row	Welch'p-value with
1e4/1e5/1e6/1e7 permutation	Average of testing
classification errors with 1e4 resampling	

Once score	
hsa-let-7d-v2-precNo2	hsa-mir-210-prec	10	215	0.0000022	0.2725975	40	
hsa-mir-210-prec	hsa-mir-30c-2No1	215	255	0.0000022	0.276602917	40	
hsa-mir-210-prec	hsa-mir-30c-1No1	215	253	0.0000022	0.284869583	40	
hsa-mir-29b-1No1	hsa-mir-338No1	246	279	0.0000023	0.282777083	40	
hsa-mir-128a-precNo2	hsa-mir-210-prec	116	215	0.0000024	0.28804875	35	
hsa-mir-185-precNo1	hsa-mir-194-precNo1	173	186	0.0000027	0.290805417	35	
hsa-mir-017-precNo1	hsa-mir-210-prec	35	215	0.0000027	0.292730833	35	
hsa-mir-210-prec	hsa-mir-26a-2No1	215	242	0.0000029	0.290358333	35	
hsa-mir-135a-2No1	hsa-mir-215-precNo2	135	223	0.0000041	0.281615417	35	
hsa-mir-215-precNo2	hsa-mir-371No1	223	297	0.000005	0.28256875	35	
hsa-mir-099b-prec-19No1	hsa-mir-124a-2-prec	79	104	0.0000063	0.28740125	35	
hsa-mir-215-precNo2	hsa-mir-3p21-v3/v4-s	e 223	319	0.0000025	0.292840833	30	
hsa-mir-138-2-prec	hsa-mir-215-precNo2	139	223	0.0000039	0.2952725	30	
hsa-mir-031-prec	hsa-mir-192-2/3No1	64	179	0.0000042	0.290688333	30	
hsa-mir-194-precNo1	hsa-mir-206-precNo2	186	213	0.0000048	0.290789167	30	
hsa-mir-29b-1No1	hsa-mir-324No2	246	267	0.0000077	0.286115833	30	
hsa-mir-192-2/3No2	hsa-mir-210-prec	180	215	0.000003	0.2987875	25	
hsa-mir-215-precNo1	hsa-mir-338No1	222	279	0.0000036	0.298490833	25	
hsa-mir-030b-precNo1	hsa-mir-210-prec	60	215	0.0000042	0.296081667	25	
hsa-mir-181a-precNo1	hsa-mir-194-precNo1	162	186	0.0000064	0.29588125	25	
hsa-mir-210-prec	hsa-mir-3p21-v3/v4-s	e 215	319	0.00001	0.2578425	25	
hsa-mir-099b-prec-19No1	hsa-mir-1-2No1	79	123	0.00001	0.26881375	25	
hsa-mir-210-prec	hsa-mir-215-precNo2	215	223	0.000001	0.300018333	21	
hsa-mir-194-precNo1	hsa-mir-210-prec	186	215	0.0000017	0.301220417	21	
hsa-mir-031-prec	hsa-mir-215-precNo1	64	222	0.0000028	0.3019975	21	
hsa-mir-030c-prec	hsa-mir-210-prec	62	215	0.0000029	0.300470417	21	
hsa-mir-181b-2No2	hsa-mir-192No1	167	181	0.0000039	0.300370417	21	
hsa-mir-181b-2No2	hsa-mir-192-2/3No1	167	179	0.000012	0.283465417	21	
hsa-mir-135a-2No1	hsa-mir-210-prec	135	215	0.000013	0.278697083	21	
hsa-mir-210-prec	hsa-mir-335No1	215	276	0.0000051	0.299356667	20	
hsa-mir-210-prec	hsa-mir-374No1	215	304	0.0000074	0.29463375	20	
hsa-mir-196-1-precNo2	hsa-mir-210-prec	189	215	0.000011	0.287699167	20	
hsa-mir-210-prec	hsa-mir-338No1	215	279	0.000011	0.29201	20	
hsa-mir-138-2-prec	hsa-mir-194-precNo1	139	186	0.0000053	0.30089	16	
hsa-mir-030c-prec	hsa-mir-138-2-prec	62	139	0.0000055	0.29982125	16	
hsa-mir-148-prec	hsa-mir-210-prec	150	215	0.0000058	0.301166667	16	
hsa-mir-148aNo1	hsa-mir-210-prec	147	215	0.0000064	0.30201625	16	
hsa-mir-1-2No1	hsa-mir-210-prec	123	215	0.0000067	0.299807917	15	
hsa-mir-206-precNo2	hsa-mir-215-precNo2	213	223	0.000007	0.299052917	15	
hsa-let-7d-v2-precNo2	hsa-mir-211-precNo2	10	216	0.0000085	0.29706375	15	
hsa-mir-185-precNo2	hsa-mir-372No1	174	299	0.0000086	0.29773375	15	
hsa-mir-206-precNo2	hsa-mir-29b-2=102pre	213	248	0.0000099	0.2962225	15	
hsa-mir-105-2No1	hsa-mir-181a-precNo1	91	162	0.0000081	0.300955833	11	
hsa-mir-019b-2-prec	hsa-mir-210-prec	39	215	0.000013	0.295918333	11	
hsa-mir-192-2/3No1	hsa-mir-338No1	179	279	0.0000092	0.299200833	10	


hsa-mir-195-prec	hsa-mir-210-prec	187	215	0.000011	0.298782917	10	
hsa-mir-124a-2-prec	hsa-mir-326No1	104	270	0.000012	0.298685833	6	
hsa-mir-030d-precNo2	hsa-mir-210-prec	63	215	0.000012	0.299224167	6	
hsa-mir-027a-prec	hsa-mir-210-prec	52	215	0.000012	0.300053333	2	
hsa-let-7g-precNo1	hsa-mir-210-prec	15	215	0.000013	0.301441667	2	


Supplementary Table S4.  miRNAs selected using different methods	
RCE	RF	Our method	
hsa-let-7a-1-prec	hsa-mir-001b-1-prec1	hsa-let-7d-v2-precNo2	
hsa-mir-030a-precNo2	hsa-mir-030b-precNo2	hsa-mir-017-precNo1	
hsa-mir-096-prec-7No2	hsa-mir-130bNo1	hsa-mir-030b-precNo2	
hsa-mir-099b-prec-19No2	hsa-mir-098-prec-X	hsa-mir-030c-prec	
hsa-mir-106bNo1	hsa-mir-330No2	hsa-mir-031-prec	
hsa-mir-129-2No1	hsa-mir-188-prec	hsa-mir-099b-prec-19No1	
hsa-mir-1-2No2	hsa-mir-101-1No2	hsa-mir-124a-2-prec	
hsa-mir-135-2-prec	hsa-mir-345No2	hsa-mir-128a-precNo2	
hsa-mir-150-prec	hsa-mir-027a-prec	hsa-mir-1-2No1	
hsa-mir-155-prec	hsa-mir-125b-2-precNo2	hsa-mir-135a-2No1	
hsa-mir-181a-precNo1	hsa-mir-194-2No1	hsa-mir-138-2-prec	
hsa-mir-185-precNo1	hsa-mir-148-prec	hsa-mir-149-prec	
hsa-mir-213-precNo1	hsa-mir-026a-precNo1	hsa-mir-181a-precNo1	
hsa-mir-214-prec	hsa-mir-324No2	hsa-mir-181b-2No2	
hsa-mir-335No2	hsa-mir-339No2	hsa-mir-185-precNo1	
hsa-mir-342No1	hsa-mir-193-precNo2	hsa-mir-185-precNo2	
hsa-mir-3p21-v1/v2-AntiS3P	hsa-mir-210-prec	hsa-mir-192-2/3No1	
hsa-mir-3p21-v3/v4-AntiS23P	hsa-mir-185-precNo2	hsa-mir-194-precNo1	
¡¡	hsa-mir-3p21-v3/v4-AntiS13P	hsa-mir-206-precNo2	
¡¡	hsa-mir-030b-precNo1	hsa-mir-210-prec	
¡¡	hsa-mir-368No1	hsa-mir-212-precNo1	
¡¡	hsa-mir-30eNo1	hsa-mir-215-precNo1	
¡¡	hsa-mir-030d-precNo2	hsa-mir-215-precNo2	
¡¡	hsa-mir-373No2	hsa-mir-26a-2No1	
¡¡	hsa-mir-103-prec-5=103-1	hsa-mir-29b-1No1	
¡¡	hsa-mir-096-prec-7No2	hsa-mir-30c-1No1	
¡¡	hsa-mir-135a-2No1	hsa-mir-30c-2No1	
¡¡	hsa-mir-340No2	hsa-mir-323No2	
¡¡	hsa-mir-135a-1No1	hsa-mir-324No2	
¡¡	hsa-mir-202-prec	hsa-mir-326No1	
¡¡	¡¡	hsa-mir-326No2	
¡¡	¡¡	hsa-mir-338No1	
¡¡	¡¡	hsa-mir-346No1	
¡¡	¡¡	hsa-mir-371No1	
¡¡	¡¡	hsa-mir-374No1	
¡¡	¡¡	hsa-mir-3p21-v1/v2-sense5P	
¡¡	¡¡	hsa-mir-3p21-v3/v4-sense45P	

Supplementary Table S5.	KEGG pathways corresponding to target union from miR-210 and miR-30c
Term	Count		%		PValue	Genes   st TotPop Hits Pop Total d Enrichm   Bonferroni	 Benjamini		FDR hsa04120:Ubiquitin mediated proteolysis		40	0.014  2.91E-11 SYVN1, XI  623	137	6910	3.23839		7.85E-09		7.85E-09	3.82E-08 hsa04115:p53 signaling pathway		20	0.007  3.99E-06 STEAP3, C 623	 67	6910	3.31089	0.00107748		5.39E-04	 0.00525 hsa05200:Pathways in cancer		62	0.022  1.19E-05 GNA13, E  623	393	6910	 1.7498	0.00321317	 0.0010722	 0.01567 hsa04114:Oocyte meiosis		25	0.009  2.72E-05 PPP2R1B,  623	109	6910	2.54392	0.00732638	0.00183665	  0.0358 hsa04141:Protein processing in endoplasmic reticulum		33	0.012  3.54E-05 RAD23B, T 623	169	6910	2.16579	 0.0095105	0.00190938	 0.04652 hsa04310:Wnt signaling pathway		28	 0.01   8.01E-05 WNT5B, B 623	138	6910	2.25045	0.02139345	0.00359778	 0.10525 hsa04931:Insulin resistance		23	0.008  1.97E-04 PPARA, IR 623	108	6910	2.36208	0.05191391	0.00758678	 0.25927 hsa03040:Spliceosome		26	0.009  2.81E-04 SRSF1, NC 623	133	6910	2.16826	0.07318842	0.00945564	 0.36944 hsa05210:Colorectal cancer		15	0.005  9.78E-04 MAP2K1,  623	 62	6910	2.68343	0.23223701	0.02893687	 1.27869 hsa05161:Hepatitis B		26	0.009   0.00107  YWHAZ, E 623	145	6910	1.98882	0.25113924	0.02850603	 1.39845 hsa04350:TGF-beta signaling pathway		18	0.006   0.00111  PPP2R1B,  623	 84	6910	2.37675	0.25832575	0.02680202	 1.44474 hsa04110:Cell cycle		23	0.008   0.00143  CDK1, E2F 623	124	6910	2.05729	0.32031115	0.03166452	 1.86272 hsa05212:Pancreatic cancer		15	0.005   0.00159  E2F3, MA  623	 65	6910	2.55958	0.34998372	0.03259226	 2.07581 hsa04914:Progesterone-mediated oocyte maturation		18	0.006   0.00166  CDK1, HS   623	 87	6910	2.29479	0.36148027	0.03153511	 2.16086 hsa04068:FoxO signaling pathway		24	0.009   0.00178  IRS2, MAP 623	134	6910	1.98654	0.38144023	0.03151676	 2.31206 hsa05166:HTLV-I infection		38	0.014   0.00243  XPO1, E2F 623	256	6910	1.64639	0.48155844	0.04022654	  3.1484 hsa05203:Viral carcinogenesis		32	0.012   0.00265  YWHAZ, IL 623	205	6910	1.73135	0.51091775	0.04119929	 3.42296 hsa04152:AMPK signaling pathway		22	0.008   0.00267  PPP2R1B,  623	122	6910	2.00011	0.51399158	0.03929219	 3.45261 hsa03015:mRNA surveillance pathway		18	0.006   0.00275  PPP2R1B,  623	 91	6910	2.19392	0.52412819	0.03833062	 3.55165 hsa05205:Proteoglycans in cancer		31	0.011   0.00348  WNT5B, R 623	200	6910	1.71918	0.61006653	0.04599747	 4.48259 hsa04520:Adherens junction		15	0.005		0.0038   NLK, TGFB 623	 71	6910	2.34327	0.64248464	0.04779969	 4.88547 hsa04390:Hippo signaling pathway		25	0.009   0.00407  YWHAZ,	623	151	6910	1.83634	0.66757253	0.04882823	 5.22187 hsa04810:Regulation of actin cytoskeleton		32	0.012   0.00414  GNA13, SS 623	211	6910	1.68212	0.67346091	0.04749613	 5.30432 hsa04510:Focal adhesion		31	0.011		0.0054   XIAP, IGF   623	206	6910	1.66911	0.76825889	0.05910363	 6.87256 hsa04380:Osteoclast differentiation		22	0.008		0.0063   FOSL2, MA623	131	6910	1.86269	0.81855426	0.06599356	 7.97553 hsa04550:Signaling pathways regulating pluripotency		23	0.008   0.00666  SMARCAD 623	140	6910	1.82217	 0.8355975	0.06708381	  8.4165 hsa05168:Herpes simplex infection		28	 0.01	0.00679  SRSF1, TB  623	183	6910	1.69706	0.84095615	0.06582863	 8.56417 hsa05222:Small cell lung cancer		16	0.006		0.0082   COL4A2, E 623	 85	6910	2.08781	0.89158446	 0.0762829	 10.2546

hsa05216:Thyroid cancer                                                          8      0.003   0.01248  CCDC6, C   623      29         6910      3.05972   0.96631256    0.11034211   15.2202 hsa00310:Lysine degradation                                                 11     0.004   0.01601  KMT2D, K 623      52         6910      2.34628   0.98720243    0.13522277   19.1234 hsa04710:Circadian rhythm                                                      8      0.003   0.01797  CSNK1E, B 623      31         6910      2.86232   0.99253155    0.14612453   21.2169 hsa04662:B cell receptor signaling pathway                        13     0.005   0.01903  BCL10, VA 623      69         6910       2.0897     0.99441849    0.14967296   22.3263 hsa05169:Epstein-Barr virus infection                                   27      0.01    0.01969  XPO1, YW 623     190        6910      1.57616   0.99534063    0.15014813   23.0064 hsa04210:Apoptosis                                                                  12     0.004   0.02108  CASP3, TN 623      62         6910      2.14674   0.99682592    0.15565841   24.4322 hsa05142:Chagas disease (American trypanosomiasis)     17     0.006   0.02287  PPP2R1B,  623     104        6910      1.81303   0.99806101     0.1634302     26.2243 hsa04919:Thyroid hormone signaling pathway                   18     0.006    0.0256   MAP2K1,  623     114        6910      1.75129   0.99909057    0.17676955   28.8947 hsa05211:Renal cell carcinoma                                               12     0.004   0.02909  CUL2, EP3 623      65         6910      2.04766   0.99965432    0.19378553   32.1665 hsa04320:Dorso-ventral axis formation                                 7      0.003   0.02989  NOTCH2,   623      27         6910      2.87557   0.99972324    0.19393292     32.897 hsa04340:Hedgehog signaling pathway                                 7      0.003   0.02989  CSNK1A1, 623      27         6910      2.87557   0.99972324    0.19393292     32.897 hsa04150:mTOR signaling pathway                                       11     0.004   0.03253  PDPK1, RP 623      58         6910      2.10356   0.99986768    0.20465641   35.2655 hsa05145:Toxoplasmosis                                                         18     0.006   0.03445  XIAP, LDL  623     118        6910      1.69192   0.99992249    0.21071402   36.9296 hsa04360:Axon guidance                                                         19     0.007    0.0348   PLXNC1, N 623     127        6910      1.65936   0.99992968    0.20802787   37.2278 hsa04144:Endocytosis                                                              33     0.012   0.03762  CHMP3, L  623     258        6910      1.41868   0.99996812     0.2184788     39.6002 hsa04151:PI3K-Akt signaling pathway                                   42     0.015   0.03779  YWHAZ, P 623     345        6910      1.35027   0.99996959    0.21484637   39.7387 hsa04071:Sphingolipid signaling pathway                            18     0.006   0.03963  PPP2R1B,  623     120        6910      1.66372   0.99998187    0.21974843   41.2382 hsa04910:Insulin signaling pathway                                      20     0.007   0.03981  IRS2, MAP 623     138        6910      1.60746   0.99998276    0.21630335   41.3807 hsa04921:Oxytocin signaling pathway                                  22     0.008   0.04432  MAP2K1,  623     158        6910      1.54439   0.99999517    0.23362626   44.9014 hsa00512:Mucin type O-Glycan biosynthesis                        7      0.003   0.05485  GALNT3, S 623      31         6910      2.50453   0.99999976    0.27681694   52.3729 hsa04066:HIF-1 signaling pathway                                         15     0.005    0.0549   MAP2K1,  623      98         6910      1.69768   0.99999976     0.2721138     52.4029 hsa03420:Nucleotide excision repair                                      9      0.003   0.05636  POLD3, RA 623      47         6910       2.1239     0.99999984    0.27357873   53.3582 hsa03013:RNA transport                                                          23     0.008   0.05743  XPOT, CL   623     172        6910      1.48316   0.99999988    0.27341206   54.0528 hsa04015:Rap1 signaling pathway                                         27      0.01    0.05783  GNAI2, CS 623     210        6910      1.42605     0.9999999     0.27047752   54.3068 hsa04922:Glucagon signaling pathway                                 15     0.005   0.05891  PKM, PPA 623      99         6910      1.68053   0.99999992    0.27040218   54.9914 hsa05223:Non-small cell lung cancer                                    10     0.004   0.06074  RASSF5, P 623      56         6910      1.98062   0.99999996    0.27329428   56.1292 hsa00520:Amino sugar and nucleotide sugar metabolis    9      0.003   0.06255  PGM3, GN 623      48         6910      2.07965   0.99999997    0.27599255   57.2264 hsa05214:Glioma                                                                       11     0.004   0.06373  IGF1R, E2  623      65         6910      1.87702   0.99999998    0.27621762   57.9288 hsa05132:Salmonella infection                                               13     0.005   0.06684  PFN1, PFN 623      83         6910      1.73722   0.99999999    0.28361854   59.7306 hsa05231:Choline metabolism in cancer                              15     0.005   0.06752  MAP2K1,  623     101        6910      1.64725   0.99999999    0.28188741   60.1131


hsa04022:cGMP-PKG signaling pathway	22	0.008	0.0685	GNA13, I	R 623	166	6910	1.46996	1	0.2813073	60.6615	
hsa04720:Long-term potentiation	11	0.004	0.06936	RPS6KA3,	623	66	6910	1.84858	1	0.28033103	61.1379	
hsa05219:Bladder cancer	8	0.003	0.07082	E2F3, CC	N 623	41	6910	2.16419	1	0.28144675	61.9285	
hsa04660:T cell receptor signaling pathway	15	0.005	0.07692	BCL10, V	A 623	103	6910	1.61527	1	0.29831936	65.0895	
hsa05162:Measles	18	0.006	0.0875	TBK1, CD	623	133	6910	1.5011	1	0.32886278	70.0009	
hsa04668:TNF signaling pathway	15	0.005	0.09255	MAP3K7,	623	106	6910	1.56955	1	0.34045761	72.1096	
hsa05215:Prostate cancer	13	0.005	0.09473	CCNE2, IG	623	88	6910	1.63852	1	0.34286691	72.9792	
hsa04920:Adipocytokine signaling pathway	11	0.004	0.09497	PPARA, IR	623	70	6910	1.74295	1	0.33932708	73.072	

Supplementary Table S6.	KEGG pathways corresponding to target union from miR-338 and miR-29b
Term	Count		%	 PValue	   Genes	List Total  Pop Hits  Pop Total d EnrichmBonferron Benjamini		   FDR hsa04510:Focal adhesion		48	0.01883   2.51E-09  CAV2, TLN	638	206	6910	2.52366   6.85E-07   6.85E-07  3.30E-06 hsa04110:Cell cycle		32	0.01255   1.60E-07  CDC14B,		638	124	6910	2.79502   4.36E-05   2.18E-05  2.10E-04 hsa05210:Colorectal cancer		21	0.00824   3.28E-07  MSH6, CY		638	 62	6910	3.66847   8.95E-05   2.98E-05  4.32E-04 hsa04151:PI3K-Akt signaling pathway		61	0.02393   7.05E-07  OSMR, FO		638	345	6910	  1.915	1.92E-04   4.81E-05  9.28E-04 hsa05161:Hepatitis B		32	0.01255   5.94E-06  MAVS, IFI		638	145	6910	2.39023	 0.00162	3.24E-04   0.00783 hsa05200:Pathways in cancer		64	0.02511   6.41E-06  STAT5A, L		638	393	6910	1.76378	 0.00175	2.92E-04   0.00845 hsa05166:HTLV-I infection		46	0.01805   1.38E-05  TLN1, NRP	638	256	6910	1.94615	 0.00376	5.37E-04   0.01815 hsa05222:Small cell lung cancer		22	0.00863   1.96E-05  COL4A2,		638	 85	6910	2.80325	 0.00534	6.69E-04   0.02581 hsa04115:p53 signaling pathway		19	0.00745   2.27E-05  CYCS, TP5		638	 67	6910	 3.0714	 0.00616	6.87E-04   0.02983 hsa05215:Prostate cancer		22	0.00863   3.44E-05  RELA, TP5		638	 88	6910	2.70768	 0.00935	9.39E-04   0.04533 hsa05203:Viral carcinogenesis		38	0.01491   4.50E-05  STAT5A, N	638	205	6910	2.00765	 0.01222	 0.00112	0.05929 hsa05205:Proteoglycans in cancer		36	0.01412   1.37E-04  CAV2, PP		638	200	6910	1.94953	  0.0368	 0.00312	0.18073 hsa05213:Endometrial cancer		15	0.00588   1.79E-04  TP53, CDH	638	 52	6910	3.12425	  0.0476	 0.00374		 0.235 hsa04512:ECM-receptor interaction		20	0.00785   2.82E-04  COL4A2,		638	 87	6910	2.48982	 0.07405	 0.00548		0.3705 hsa05169:Epstein-Barr virus infection		33	0.01295   5.33E-04  VIM, NFKB	638	190	6910	1.88113	 0.13556	 0.00966	0.70032 hsa04210:Apoptosis		15	0.00588	0.00124  RELA, CYC		638	 62	6910	2.62034	 0.28642	 0.02087	1.61489 hsa05146:Amoebiasis		21	0.00824	0.00143  COL4A2, C	638	106	6910	2.14571	 0.32262	 0.02265		1.8617 hsa04919:Thyroid hormone signaling pathw		22	0.00863	 0.0015	ACTB, ATP	638	114	6910	2.09014	 0.33676	 0.02255	1.96148 hsa04520:Adherens junction		16	0.00628	0.00172  ACTB, PTP		638	 71	6910	2.44073		0.375	 0.02443	2.24202 hsa05220:Chronic myeloid leukemia		16	0.00628	0.00199  RELA, STA		638	 72	6910	2.40683	 0.41991	 0.02686	2.59301 hsa05214:Glioma		15	0.00588		0.002	TP53, CDK		638	 65	6910	 2.4994	 0.42112	 0.0257	2.60287 hsa04068:FoxO signaling pathway		24	0.00942	0.00242  SGK1, SMA   638	134	6910	1.93983	 0.48457	 0.02968	3.14687 hsa04141:Protein processing in endoplasmi		28	0.01098	 0.0031	SEC31A, U		638	169	6910	1.79444	 0.57201	 0.03623	4.01157 hsa04917:Prolactin signaling pathway		15	0.00588	0.00471  RELA, STA		638	 71	6910	2.28818	 0.72461	 0.05231	6.03185 hsa05230:Central carbon metabolism in can		14	0.00549	0.00489  TP53, PFK		638	 64	6910	2.36922	 0.73753	 0.0521		6.2495 hsa04390:Hippo signaling pathway		25	0.00981	0.00549  ACTB, DVL	638	151	6910	1.79317	 0.77774	 0.0562	6.99852 hsa05212:Pancreatic cancer		14	0.00549	0.00561  RELA, TP5		638	 65	6910	2.33277	  0.7847	 0.05529	7.14123 hsa04974:Protein digestion and absorption		17	0.00667	0.00601  COL4A2, C	638	 88	6910	 2.0923	 0.80705	 0.05707	7.63079

hsa04010:MAPK signaling pathway                      37        0.01451    0.00603  MEF2C, Z      638           255          6910      1.57152    0.8084     0.05539    7.66226 hsa04310:Wnt signaling pathway                         23        0.00902     0.0074    CSNK1A1,     638           138          6910      1.80512   0.86823    0.06532    9.31476 hsa05206:MicroRNAs in cancer                             40        0.01569    0.00748  MCL1, EZ      638           285          6910       1.5201    0.87109    0.06395     9.4107 hsa04668:TNF signaling pathway                          19        0.00745     0.0077    RELA, CSF     638           106          6910      1.94136   0.87886    0.06383    9.68208 hsa04550:Signaling pathways regulating plu      23        0.00902    0.00875  DVL3, JAR     638           140          6910      1.77933   0.90926    0.07014    10.9322 hsa05164:Influenza A                                              27        0.01059    0.00895  MAVS, IFI      638           174          6910      1.68063   0.91408    0.06964    11.1668 hsa05162:Measles                                                   22        0.00863    0.00975  MAVS, IFI      638           133          6910      1.79155   0.93112    0.07359    12.1088 hsa05219:Bladder cancer                                       10        0.00392    0.01081  NRAS, CD      638            41           6910      2.64164   0.94861    0.07914    13.3422 hsa05218:Melanoma                                               14        0.00549    0.01191  TP53, CDH    638            71           6910      2.13564   0.96201    0.08459    14.5958 hsa04915:Estrogen signaling pathway                 17        0.00667    0.01821  GNAI2, ES     638            99           6910      1.85982   0.99337    0.12366    21.4943 hsa04810:Regulation of actin cytoskeleton        30        0.01177    0.01823  ENAH, DIA    638           211          6910      1.53991   0.99342    0.12086    21.5234 hsa05202:Transcriptional misregulation in c      25        0.00981    0.01967  CCNT2, M     638           168          6910      1.61171   0.99559    0.12681     23.024 hsa05145:Toxoplasmosis                                        19        0.00745    0.02216  GNAI2, M     638           118          6910      1.74393    0.9978     0.13863    25.5608 hsa05100:Bacterial invasion of epithelial ce       14        0.00549    0.02498  ACTB, CA      638            78           6910      1.94398      0.999      0.15161    28.3339 hsa04722:Neurotrophin signaling pathway        19        0.00745    0.02586  RELA, TP5     638           120          6910      1.71486   0.99922    0.15324    29.1835 hsa04152:AMPK signaling pathway                      19        0.00745    0.03001  SREBF1, H     638           122          6910      1.68675   0.99976    0.17223    33.0509 hsa05221:Acute myeloid leukemia                       11        0.00432    0.03017  AKT1, NRA    638            56           6910      2.12746   0.99977    0.16959    33.1987 hsa05131:Shigellosis                                                12        0.00471    0.03058  ACTB, CD      638            64           6910      2.03076   0.99979    0.16835    33.5761 hsa04340:Hedgehog signaling pathway                7         0.00275    0.03313  CSNK1A1,     638            27           6910      2.80796    0.9999     0.17773    35.8345 hsa05211:Renal cell carcinoma                             12        0.00471    0.03388  AKT1, CD       638            65           6910      1.99952   0.99992      0.178      36.4869 hsa04932:Non-alcoholic fatty liver disease (      22        0.00863    0.03615  SREBF1, C     638           151          6910      1.57799   0.99996    0.18546    38.4261 hsa04150:mTOR signaling pathway                      11        0.00432    0.03749  EIF4B, AKT    638            58           6910       2.0541    0.99997    0.18829    39.5422 hsa05231:Choline metabolism in cancer             16        0.00628    0.04277  SLC44A2,      638           101          6910      1.71576   0.99999    0.20865    43.7732 hsa05216:Thyroid cancer                                         7         0.00275    0.04548  NRAS, CCN   638            29           6910      2.61431          1          0.21682    45.8331 hsa05168:Herpes simplex infection                      25        0.00981    0.04809  MAVS, SR     638           183          6910      1.47961          1           0.2242     47.7486 hsa01130:Biosynthesis of antibiotics                   28        0.01098    0.05145  TM7SF2, L    638           212          6910      1.43047          1          0.23434    50.1235 hsa04914:Progesterone-mediated oocyte m     14        0.00549     0.0544    CCNB1, AK    638            87           6910      1.74287          1          0.24245    52.1343 hsa05160:Hepatitis C                                               19        0.00745    0.06202  MAVS, RN     638           133          6910      1.54725          1          0.26813    56.9731 hsa05217:Basal cell carcinoma                              10        0.00392    0.06242  SMO, DVL     638            55           6910      1.96922          1          0.26559    57.2104 hsa04066:HIF-1 signaling pathway                       15        0.00588    0.06449  RELA, PD       638            98           6910      1.65776          1          0.26932    58.4392


hsa05223:Non-small cell lung cancer	10	0.00392	0.06859	AKT1, NR	638	56	6910	1.93406	1	0.28022	60.7773	
hsa04931:Insulin resistance	16	0.00628	0.0697	SREBF1, P	638	108	6910	1.60455	1	0.28015	61.3836	
hsa04350:TGF-beta signaling pathway	13	0.0051	0.08276	ACVR2B,	638	84	6910	1.67618	1	0.32066	67.9482	
hsa04611:Platelet activation	18	0.00706	0.08793	ACTB, TL	N	638	130	6910	1.49964	1	0.33321	70.2476	
hsa04662:B cell receptor signaling pathway	11	0.00432	0.09946	AKT1, NR	638	69	6910	1.72664	1	0.36491	74.8379	


Supplementary Figure S1. A hierarchical clustering with complete linkage and centered Pearson correlation after z-score transformation of linear expression projections for individual significant miRNAs.
